# Refining Convergent Rate Analysis with Topology in Mammalian Longevity and Marine Transitions

**DOI:** 10.1093/molbev/msab226

**Published:** 2021-07-29

**Authors:** Stephen Treaster, Jacob M Daane, Matthew P Harris

**Affiliations:** 1 Department of Orthopaedic Research, Boston Children’s Hospital, Boston, MA, USA; 2 Department of Genetics, Harvard Medical School, Boston, MA, USA; 3 Department of Marine and Environmental Sciences, Northeastern University Marine Science Center, Nahant, MA, USA

**Keywords:** mammalian longevity, marine transition, comparative genomics, convergence

## Abstract

The quest to map the genetic foundations of phenotypes has been empowered by the modern diversity, quality, and availability of genomic resources. Despite these expanding resources, the abundance of variation within lineages makes it challenging to associate genetic change to specific phenotypes, without an a priori means of isolating the changes from background genomic variation. Evolution provides this means through convergence—that is, the shared variation that may result from replicate evolutionary experiments across independent trait occurrences. To leverage these opportunities, we developed *TRACCER: Topologically Ranked Analysis of Convergence via Comparative Evolutionary Rates*. Compared to current methods, this software empowers rate convergence analysis by factoring in topological relationships, because genetic variation between phylogenetically proximate trait changes is more likely to be facilitating the trait. Comparisons are performed not with singular branches, but with the complete paths to the most recent common ancestor for each pair of lineages. This ensures that comparisons represent a single context diverging over the same timeframe while obviating the problematic requirement of assigning ancestral states. We applied TRACCER to two case studies: mammalian transitions to marine environments, an unambiguous collection of traits that have independently evolved three times; and the evolution of mammalian longevity, a less delineated trait but with more instances to compare. By factoring in topology, TRACCER identifies highly significant, convergent genetic signals, with important incongruities and statistical resolution when compared to existing approaches. These improvements in sensitivity and specificity of convergence analysis generate refined targets for downstream validation and identification of genotype–phenotype relationships.

## Introduction

When challenged with similar selective pressures, independent lineages may converge on similar adaptations to those challenges (Losos 2011). A myriad of mutations may be sufficient to produce such adaptive traits, but considering the constraints and largely similar toolkits of life (Rosenblum et al. 2014), the stochastic nature of evolution means that such adaptive traits will tend to manifest parsimoniously. Given the developmental, physiological, and genetic constraints across organisms, the convergent evolution of traits will therefore tend towards similar molecular mechanisms. Genetic similarities in trait-sharing lineages, beyond those expected by chance, can provide a map for phenotype-to-genotype inferences, recently coined as “forward genomics” (Hiller et al. 2012; Currie 2013). While the parsimonious path to the same adaptive phenotype may tread upon the same genetic systems, the specific molecular changes may evolve in unique ways. The molecular similarities underlying convergent traits will be shaped by both the broader genetic context and the degrees of freedom in the mechanisms involved; there are countless ways to disrupt a pathway or knock out a gene, but there may only be a handful to specifically compromise a hydrophobic pocket, and only one to create a disulfide bridge between two domains. A variety of changes could modulate cellular activity in comparable ways and ultimately manifest as convergent phenotypes of interest. Analytical tools with a general measure are needed to capture these disparate genetic signatures acting on the same system in order to make meaningful comparisons between species.

### Relative Evolutionary Rates and Convergence

The assessment of relative evolutionary rates (RERs) has arisen as a powerful and flexible way to distill and compare variable genomic signatures to identify convergence. RERs describe the degree of change in a genomic region as compared to the background rate across that genome. Not all parts of a genome evolve at the same pace, so these relative rates can vary widely. While mutations are randomly distributed, finely tuned core pathways will undergo strong purifying selection, constraining sequence identity, and leaving loci relatively unchanged across hundreds of millions of years (Katzman et al. 2007). These loci will reliably yield low RERs. In contrast, high RERs indicate either positive selection facilitating adaptation or neutral selection when functionality is no longer integral. These occur in the “wilderness” between functional loci, where mutations can accumulate without strong selective pressure. Rates in these regions can vary (Palazzo and Gregory 2014) but in principle yield higher RERs. Duplicated and pseudogenized regions, also freed from restriction, can neofunctionalize to facilitate adaptation or go to drift and thus may also yield high RERs. These accelerations and constraints can be powerfully informative as to the selective pressures acting on a locus (Pollard et al. 2006; Echave et al. 2016). Importantly, RER analyses can scale in scope, from single positions to exons, genes, or even to discontiguous loci comprising entire pathways (Daane et al. 2020). This scalability contrasts with analyses that quantify a specific measure, like gene loss, expansion, or the detection of identical amino acid substitutions. The last, for instance, has proven informative in many systems, such as echolocation (Parker et al. 2013), but their relevance remains contentious (Zou and Zhang 2015; Marcovitz et al. 2019). Such specific measures alone fail to capture local, potentially linked, changes that could influence the trait similarly. Single amino acid substitutions are too narrow a measure to encompass the multiple dimensions molecular evolution can act upon. RERs scale to the degree of specificity an experiment may require and can capture disparate yet functionally comparable changes in a single measure.

**Fig. 1.**
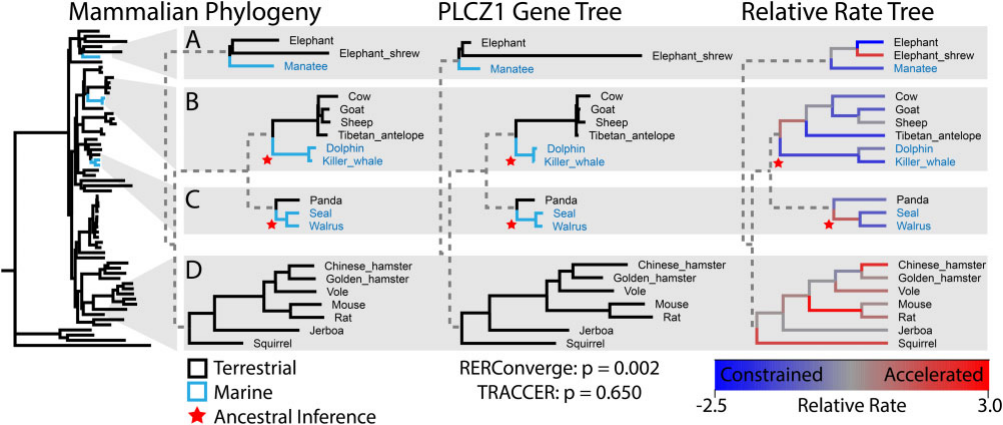
Relative evolutionary rates, topology, and ancestral state assignments in convergence analyses. A. Rates of genome-wide variation (the phylogenetic tree) are compared to gene-specific rates to calculate RERs. For example, the Elephant and Elephant Shrew terminal branches of gene *PLCZ1* are shorter and longer respectively than their background genomic rates, yielding strong constraint (blue) and acceleration (red) signals on the RER Tree. *PLCZ1* shows constrained evolution on the terminal branches of all five marine mammals (*A–C*) and conventional statistics indicates a significant association between *PLCZ1* evolutionary rate and marine transitions (RERConverge *P* = 0.002). However, most of the terrestrial relatives closest to the marine lineages demonstrate similar constraint at this locus (e.g., elephant, antelope and panda; *A–C*, respectively), suggesting PLCZ1 constraint is a not specific for marine transitions, but a shared property of the broader clades. (*C*) Previous analyses delineate the ancestral pinniped as terrestrial, though it is likely marine. As 1/7th of the marine branches, its acceleration signal (red) would undermine the convergent constraint shared by the marine terminal branches. If either the seal or walrus was missing, thereby merging the ancestral and remaining terminal branch, the pinniped group would have yielded an acceleration signal. (*D*) The collection of rodents is consistently accelerated, encompassing a large percentage of the branches on the tree and anchoring the RER distribution, but is distantly related from the marine mammals. By treating every branch as an equivalent and independent instance of marine and terrestrial evolution, conventional methods yield an arguably spurious convergence signal (*P* = 0.002). TRACCER considers that closely related sister lineages are also constrained for this locus, often more so than the marine lineages, while distal clades with no trait change represent much of the acceleration signal. Without needing to infer ancestral states, TRACCER assigns *PLCZ1* a nonsignificant score (*P* = 0.65).

RERs for a single element (exon, gene, CNE, etc.) across a group are generally represented with phylogenetic trees, and the relative rates can be derived by comparing individual gene trees to the species tree. For example, *PLCZ1*, a phospholipase that triggers calcium fluctuations during sperm-egg recognition, has a much shorter branch length in elephants when compared to the average across its genome, resulting in a low RER. In contrast, the Elephant Shrew branch is longer than expected and yields a high RER (fig. 1*A*). To identify convergence in rate, the RERs for a specific element can be compared among species and intersected with trait occurrence. Lineages sharing both traits and RERs, or those with diverging traits and differing RERs, are evidence that the locus may be involved in the evolution of that trait. With tens of thousands of loci each deriving their own RER, a distribution of acceleration and constraint would be expected, centered on the background substitution rate. By analyzing many comparisons across multiple instances of trait change, consistently shared RERs for a specific element are unlikely to occur, enabling detection of convergent signals from genomic noise. As RERs are a continuous variable, calculations and statistics are also empowered by including their magnitude.

Until recently, tools for RER analysis were limited to individual loci or specific branches and did not scale to the modern wealth of genomic information. The first broadscale “forward genomics” approach used a “Perfect Match” measure of shared declines in percent identity in conjunction with trait loss to implicate regions in the regulation of that trait. It was agnostic regarding topology and did not compute significance but identified *Gulo* as drifting with the loss of vitamin C synthesis and *Abcb4* as drifting with low biliary phospholipid levels (Hiller et al. 2012). The RERConverge tool later efficiently analyzed RER convergence in both acceleration and constraint, implicating genes in marine mammal transitions (Chikina et al. 2016), mammalian subterranean transitions (Partha et al. 2017), and mammalian longevity (Kowalczyk et al. 2020) with revealing insights into the genetic architecture that facilitates these dramatic environmental and physiological shifts. The “Perfect Match” approach was also refined with two new methods (Prudent et al. 2016): the “Generalized Least Squares” method corrects for phylogenetic relatedness but has many assumptions (Mundry 2014) does not compensate for sampling distribution, and only detects drift related to trait loss; and the “Branch Method” is very much akin to RERConverge. Both RERConverge and the “Branch Method” assume each branch is an equivalent and independent occurrence of evolution, and as such, process the signals with conventional statistical tests that require independence, including the Pearson Correlation or Wilcoxon Rank-Sum. However, these assumptions are incongruent with the conceptual basis of convergence and have consequences for both sensitivity and specificity.

### The Value of Topology

Not all lineage comparisons are equally informative, nor are they truly independent; topology and genetic context must be factored in. Sister lineages with differing traits make for more valuable and informative comparisons than distant ones: the changes facilitating the trait occurred within a shorter-time frame and with a greater degree of shared genetic architecture. There is less variation between sister lineages compared to more distant comparisons, such that any differences revealed are more likely to be facilitating the trait difference being investigated. The opposite is equally important: when closely related species with differing traits share a genetic signature, it is strong evidence that the signature is unrelated to the trait. Regardless of the arguments dividing convergence and parallelism (Gould 2002; Pearce 2012), neglecting topology is an oversight of many statistical approaches to associate RERs with trait change. An example of these effects is highlighted in figure 1. There, RERConverge has assigned *PLCZ1* a significant score for convergent constraint in the evolution of marine mammals (fig. 1*A–C*). However, the closest terrestrial relatives all share similar levels of constraint, suggesting that constrained *PLCZ1* evolution is not specific for marine transitions, but instead a general signature of the larger clades. In contrast, the rodents (fig. 1*D*) are enriched for accelerations in *PLCZ1*, represent a substantial percentage of the branches on the tree, yet are distantly related from any marine lineages. The available mammalian genomes are oversampled for many clades that have little to do with marine transitions. The disproportionate number of branches in these clades can spuriously anchor conventional statistics. Despite these branches representing variable genetic contexts, timeframes, and relationships to the trait being analyzed, conventional statistics must treat them each *as an equivalent and independent instance* of marine or terrestrial evolution. For instance, in the analysis of marine mammals, it is problematic to include the ancestral branch leading to mice and rats—roughly 100 M years from the closest marine mammal—as an equivalent comparison and weight as the panda terminal branch, the immediate sister of pinnipeds. Individual branches on a tree are not equally meaningful; they are arbitrary fragments of evolution based on which lineages were sampled, each with their own context and varying degrees of shared history. Instead, the branches from which RERs are derived and compared should represent comparable evolutionary events; specifically, divergent paths from a single context, over the same time frame.

To fully integrate the informative power present in a phylogeny into convergence analysis requires a delineation of which evolutionary events should be compared. Most convergence analyses require flagging specific branches for analysis, with often tenuous assumptions of ancestral states. This is easier with well-delineated traits, such as marine mammal adaptions, that have a well-documented fossil record (Slater et al. 2012). Misattribution of ancestral states can easily compromise an analysis. In Chikina et al. (2016), the pinniped ancestral branch was not assigned as marine, biasing the constraint signal for *PLCZ1* by leaving out an accelerated branch (fig. 1*C*). If the genome for either the seal or walrus was unavailable, the entire pinniped lineage would have been designated marine and calculated as accelerated. Instead, because the branches are arbitrarily fragmented by sampling availability, both pinniped terminal branches are generating a strong signal of purifying selection. Critically, these branches likely do not represent a marine transition at all; it is likely the ancestor to the pinniped clade that was undergoing the dramatic adaptation (Berta et al. 2018). Strictly defining the trait status of each branch manifests a potential to misattribute when selection may be acting, which can spuriously boost or abate signals of selection. This is difficult enough with a clear trait-like marine transitions (the pinniped phylogeny was contentious for some time and many argued it was diphyletic; Koretsky and Barnes 2003) and would be nigh impossible for inconspicuous traits like longevity or metabolism. This problematic requirement to assign ancestral states is common in convergence analyses. To our knowledge, current rate convergence analyses either ignore topology, or require inferring ancestral states, and/or do not scale to genome-wide data.

### New Approaches

In light of these concerns, we developed a new analysis pipeline, *Topologically Ranked Analysis of Convergence via Comparative Evolutionary Rates—TRACCER*. We test if the analysis of convergence is refined by making genomic comparisons between trait-bearing and nontrait-bearing extant species in reference to their most recent common ancestor (MRCA). With this approach, comparisons represent a single genetic context, diverging over the same time frame, and are guaranteed to encompass a change in trait because the diametric terminal states are assigned and included. This approach allows three critical features 1) comparisons can be weighted by the phyletic distance between them, such that proximal instances of trait change are given greater influence, while phylogenetically distal comparisons can be attenuated, 2) it is no longer necessary to infer ancestral states, and 3) trait distribution can be incorporated to compensate for sampling biases. In brief, for a single tree, these features are implemented by calculating each pairwise difference in RER occurring since that pair’s MRCA, then scaling the difference by their total variance (i.e., the total branch distance from one to the other on the species phylogeny). The scalar uses modified internal branch lengths that are stretched by the number of lineages that share it, thus offsetting oversampled clades with little trait change. Pairwise scores are then summed across all combinations of trait- and nontrait-bearing species to derive a gene score. The details of these calculations are elaborated in the methods section and figure 6.

By factoring in phylogenetic relatedness, we can no longer use conventional statistics to determine the significance of these gene scores, as each comparison is no longer independent. As we are comparing lineages in reference to their MRCA, many comparisons will share some ancestral branches, causing varying degrees of dependence between comparisons. Instead, we use a permutation strategy to repeatedly shuffle and sample branches of the element trees to simulate how likely a pattern of relative rates is to occur on a given topology with the actual data. The combinatorial complexity of proteome-sized data sets on a broadly sampled phylogeny is sufficient to sample from directly. Because RER calculations require a fixed topology, we can shuffle branches between trees to yield a simulated instance of evolution using real examples of how those speciation events occurred at a molecular level. Millions of these phylogenetically and molecularly grounded permutations are analyzed alongside the experimental scores to determine the background score distribution. The experimental results are compared to this distribution to determine their significance. As this distribution may not be symmetrical, accelerated genes are only compared to permutations scoring above the median, and constrained scores are only compared to those below. Missing lineages, which are common in genomic data sets, are a nonissue when comparing genes with TRACCER: each unique topology derives its own score-space and significance calculations. The permutation approach contrasts with the variety of evolutionary models one could draw from, which while undeniably useful, may generate predictions that fail to describe real data (Naser-Khdour et al. 2019). Thus, TRACCER obviates the need for assumptive formulae by drawing directly from the wealth of information inherent to modern comparative genomic data sets.

TRACCER diverges in a few key details of RER calculations from previous analyses with important ramifications. We use a log-transformed fold change from baseline, as opposed to the residual approach used by RERConverge. Residuals fail to inversely allocate weight to variation in longer branches; two SNPs in ten million years should not hold the same influence as two SNPs in one million years, yet these would calculate the same residual value. The fold change approach inherently corrects for branch length and embraces the “rate” concept of relative rates. TRACCER also uses a median distance across all gene trees, excluding zeroes, to derive the relative rates. The median calculation is robust to acceleration outliers but would be skewed by the number of zero-length branches if they were not excluded. Many evolutionary models and tree calculations often struggle with these regions that lack informative substitutions. Due to their short sequence and/or extreme selective pressure, they are guttered to zero-length branches which does not accurately represent the forces at play across the genome. These branches are still informative and should be analyzed as undergoing constraints. They can still be compared alongside the rest of the branches, as each branch within a tree will have a consistent bias. The details of these calculations are elaborated in the methods.

These analytical concepts—comparing RERs in reference to MRCAs, weighting comparisons by evolutionary distance, deriving RERs as log-fold changes from zero-corrected-medians, and calculating significance through comparison to permutations of the phylogenetic data—empower relative rate analyses to better reveal convergent signatures. TRACCER compares relative evolutionary rates in a pairwise approach that obviates the need for ancestral state assumptions and allows for topologically aware comparisons; it prioritizes sister groups with high comparative power and dilutes the influence of less informative outgroups. RERs are derived as the fold change from the corrected median branch length, accurately describing rates without branch-length bias or assumptive models. TRACCER defaults include a ranking transformation applied to signal magnitudes, preventing outlier lineages from swamping potential signals, without leaving them ignored. Finally, by scaling each comparison by how often those branches have been used, TRACCER dilutes the influence of oversampled clades and their shared ancestral branches, while allowing their unique terminal branches their full influence. Through these attributes, the recurrent concept of convergence is ensured while increasing sensitivity to subtle but meaningful changes over short timeframes.

## Results

To benchmark the impact of these strategies, we have directly compared TRACCER to a similar analysis, RERConverge, which has recently demonstrated great success analyzing marine mammal transitions and mammalian longevity. These represent two test cases of convergence. The marine mammal transition is a dramatic phenotype that is well-documented in the fossil record but has only occurred three times independently. It is an iconic test case for convergence analyses. In contrast, longevity shifts are poorly delineated ancestrally, but the trait varies across the entire mammalian phylogeny with numerous independent instances of gain and loss.

### Test Case 1: Marine Mammal Convergence

We obtained amino-acid level gene alignments for 62 mammals from the UCSC 100-Way Vertebrate Multiz data set (Rosenbloom et al. 2015). We chose this set to match previous analyses with RERConverge (Chikina et al. 2016) and allow direct comparison of statistical resolution. The species tree provided by UCSC was trimmed to maintain just the mammals and used to fix the topology of 37,272 protein-coding trees generated with PAML using the same settings as described in Chikina et al. (2016). The five marine mammals, including the cetaceans (dolphin, killer whale), pinnipeds (seal, walrus), and sirenians (manatee), represent three independent cases of adaptation to an aquatic environment (fig. 1). When using RERConverge, which requires ancestral branches to be classed, we additionally flagged the cetacean ancestor as marine, but not the pinniped ancestor, again to match published methodology and results. As a control group, we also followed the published RERConverge selections to allow for direct comparison: aardvark, alpaca, camel, little brown bat, and David’s myotis bat have roughly similar topology as the marine mammals and provide a nonconvergent control group to assess background signal levels.

**Fig. 2.**
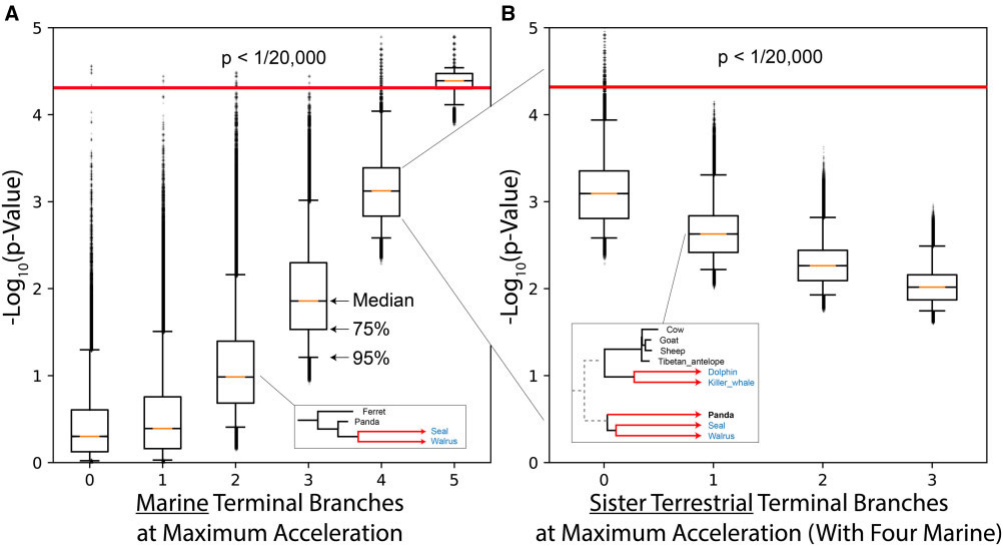
Boundaries of significance for marine mammal convergence. (*A*) To highlight how topology influences significance in TRACCER, ten million randomly simulated mammalian gene trees were scored for convergence in marine lineages. Significant probability scores less than 1/20,000 were detected, indicating the topology of the marine data set is sufficiently complex to identify convergent signals on a proteome-sized data set (*A0*, above red line). To gauge how these *P*-values were shaped, we forced random marine lineages to extreme acceleration (*A1:5*), compressing values towards the significance boundary. However, even with all five accelerated, random variation in the terrestrial lineages could produce scores below significance (*A5*). (*B*) Focusing on the four marine acceleration box and additionally accelerating immediate sister terrestrial lineages reveals how proximate comparisons can influence the signal. While four marine lineages sharing a signal is capable of yielding significant scores (*A4*), if the closest terrestrial lineage shares the same extreme acceleration (*B1*), it is sufficient to prevent those scores from reaching significance. In the example box, the accelerated Panda lineage is undermining the convergent acceleration shared by four random marine lineages.

TRACCER has an integrated diagnostic mode to assess if a particular tree topology has sufficient power to reveal convergence beyond background noise and provide an estimate of how robust of a signal is required to reach that significance. It is important to note that the maximum score for marine convergence with TRACCER is not derived from the mere fact that all marine (trait) species exhibit the same extremes of selection; their terrestrial (nontrait) sister lineages must occupy the opposite end of the distribution for that gene. Put another way, immediate sister lineages sharing the same relative rate polarity should compromise the convergent signal, as the signal is no longer specific for the trait in that genetic context. To profile these dependencies, we simulated 10,000,000 gene trees with Brownian motion (Revell et al. 2008) and measured the significance of their convergent signals against a backdrop of another 10,000,000 random trees (fig. 2*A*, “0”). This revealed that *P*-values less than 1/20,000—roughly the size of a vertebrate proteome data set—were possible, indicating the topology represented by the three marine transitions yields a sufficiently complex score-space to parse significant hits from the background noise of a genomic data set.

To gauge the shape or robustness of the convergent pattern required to produce these significant hits, the diagnostic allocates extreme rate accelerations to random terminal branches on top of the Brownian backdrop. We see that with all five marine terminal branches set to maximum acceleration, the vast majority of trees yield a significant score, but not all of them. If the immediate sister lineages were also highly accelerated, they reduce the score below the threshold (fig. 2*A*, “5”). We demonstrated this effect by again purposefully allocating extreme accelerations, this time to four marine mammals along with a number of their immediate sisters (fig. 2*B*). While setting extreme accelerations to four marine terminal branches can drive significant scores, even a single sister lineage sharing that extreme acceleration will entirely prevent it. If the three closest terrestrial lineages share the same extreme signal as four marine mammals, while all other branches are randomly distributed, it will reduce the median *P*-value by an order of magnitude. These simulations are simplified by distributing the acceleration signals only to terminal branches and randomizing all others but they provide a valuable overview of the topological weight influence.

**Fig. 4.**
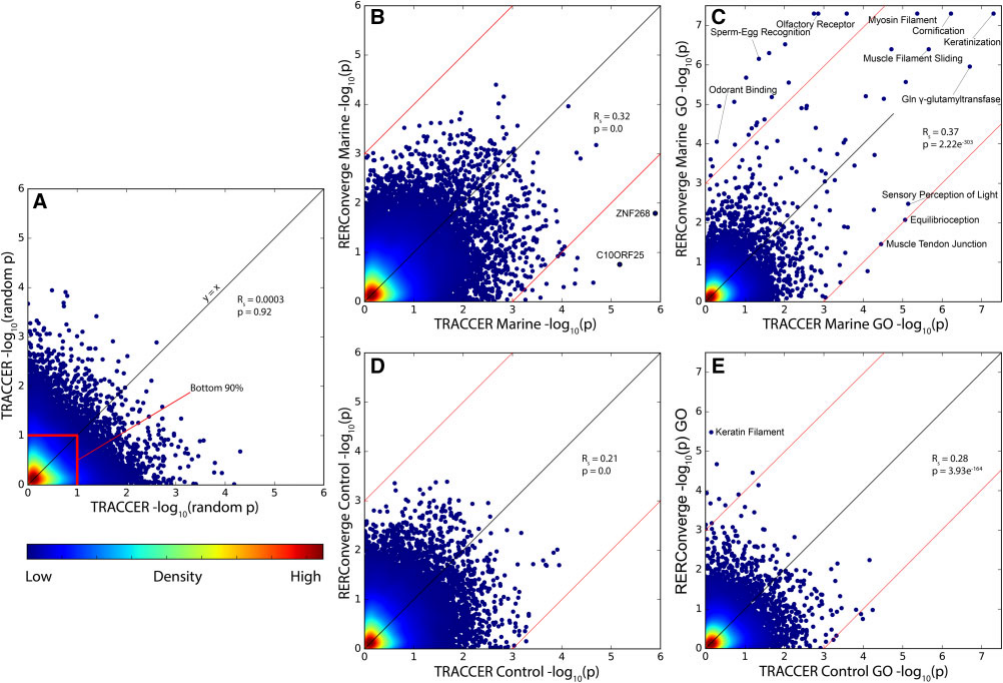
Comparison of TRACCER and RERConverge in marine mammal convergence. (*A*) Demonstration of the shape of a random log/log distribution expected by chance with a proteome sized data set using two different, nonconvergent species selections. Color indicates density of values, highlighting the shared bottom 90% of *P*-values concentrated at the origin, and no correlation in results. (*B*) Concordance between TRACCER and RERConverge is extremely significant, though TRACCER detects a number of highly significant targets that RERConverge does not (outside of red lines). (*C*) GO enrichment of TRACCER and RERConverge gene scores demonstrates agreement on many highly significant terms with established roles in marine transitions (within red lines). However, RERConverge also identifies many terms involved in olfaction and sperm morphology, though there is evidence these are unlikely to be convergently evolving with this definition of marine. (*D*) Concordance in the marine negative control data set is also highly significant, and as expected, neither has any individual genes as highly significant. (*E*) GO enrichment of the negative control data set, highlighting Keratin Filament as significantly enriched in the RERConverge results, and a lack of false positives with TRACCER.

When applied to the real mammalian genomic data set, TRACCER reveals substantial enrichment of genes at lower *P*-values, indicating convergence signals well above the flat background rate seen in the control group (fig. 3). In our use, and matching the published methods, RERConverge shows a similar *P*-value distribution and accurately recapitulates established results with that tool. Unlike those publications, in representing these results and for downstream analyses, we do not segregate accelerated and constrained genes, instead of focusing solely on significance. The concordance between these methods is high, with a Spearman correlation of 0.32 and *P*-value less than 1E−350 (fig. 4*B*). However, a number of genes are dramatically shifted between these analyses, both increasing and decreasing in statistical significance. Unlike RERConverge, many TRACCER gene scores remain significant after false discovery correction (Supplementary S1 online) indicating an improvement in sensitivity. To resolve trends in these differences, GO gene set enrichment was performed using the SUMSTAT approach, which has demonstrated power, flexibility, and simplicity over competing gene set enrichment analyses (Ackermann and Strimmer 2009; Tintle et al. 2009; Chen et al. 2010). Both TRACCER and RERConverge reveal highly significant processes and systems evolving to facilitate mammalian marine transitions (fig. 4*C*, Supplementary S2 online). They agree on many of the most significant terms, with extremely significant *P*-values for cornification, keratinization, myosin filament, and muscle filament sliding. These each have obvious implications with the transition to a marine environment, which required dramatic epithelial and muscular adaptations. Another shared, highly significant gene set, “protein-glutamine gamma-glutamyltransferase activity,” is likely playing a role in the marine metabolic and dietary adaptions, particularly with glucose regulation and has intriguing parallels with diabetes pathology in the literature (Schermerhorn 2013; Venn-Watson 2014).

**Figure:**
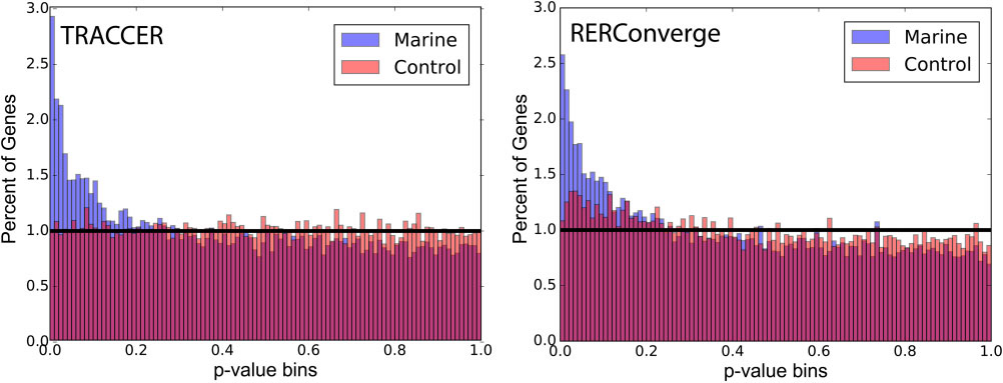
Fig. 3. Marine mammal RER convergence significance distributions. Results from both TRACCER and RERConverge analyses are dramatically enriched at low *P*-values when analyzing for marine convergence (blue). The TRACCER control distribution (red) is flat, matching the distribution expected by chance. The RERConverge control is slightly enriched at low *P*-values, suggesting branch effects.

However, when we focus on the GO-terms with the greatest shift in significance between the two analyses, we reveal some systems are differentially detected, including sperm-egg recognition and odorant binding (fig. 4*C*). Multiple terms involving sperm physiology are differentially detected as significant by RERConverge (Supplementary S2 online). As an example, the “sperm-egg recognition” gene set is one of the most dramatically shifted, with a *P*-value of 7E−07 using RERConverge, but only 0.044 from TRACCER. We earlier highlighted *PLCZ1* (fig. 1) which is the greatest contributor to the “sperm-head” signal in RERConverge. With a *P*-value shift from 0.002 to 0.65, *PLCZ1* is an excellent example of how topological weighting and ancestral inferences are underscoring the observed differences in the analyses.

TRACCER and RERConverge also differ on the conventionally marine-associated GO terms “olfactory receptor activity” and “odorant binding proteins” (ODPs). While there is plenty of evidence that olfaction in general should be reduced, it is not entirely absent, and the discrepancy in these terms may be important. It is certain that many olfactory receptors have been pseudogenized (Hayden et al. 2010), and the olfactory bulb is reduced in most cetaceans and entirely absent in toothed whales (Kishida et al. 2015). Yet, it is only modestly significant in TRACCER (*P* = 0.0014) and extremely significant in RERConverge (*P* < 1E−07). The differential detection of ODPs is driving this shift, as they are entirely a subset of “olfactory receptor activity,” and while significant with RERConverge (*P* = 8.80E−05), are not with TRACCER (*P* = 0.52).

The mechanisms and functions of vertebrate ODPs have proven puzzling, but there is evidence they are involved in the perception of pheromones more so than general olfaction (Tegoni et al. 2000). Many marine mammals still utilize pheromones and require the molecular machinery to detect them, so they are unlikely to have been universally compromised as the olfactory receptors have. For instance, dolphins likely employ and detect sex pheromones in their urine (Muraco and Kuczaj 2015). Critically, the pinnipeds still maintain their vomeronasal system, including strong purifying selective pressure on *TRPC2*, an essential genetic component (Yu et al. 2010), likely because they still maintain some terrestrial behavior. Strictly flagging the pinnipeds as marine mammals, and not their ancestral branch, despite both spending a large fraction of their life on land, may be confounding the analysis. The delineation between terrestrial and marine mammals is not as cut-and-dried as most convergence publications suggest and echoes the difficulties of inferring ancestral states. The evolution of pheromones in marine mammals and the molecular systems for their detection may be more complex than originally assumed, but according to TRACCER the “odorant binding” gene set is not convergently evolving with this selection of marine mammals, thereby limiting the significance of the more general “olfactory receptor activity” superset.

As a negative control for the gene set analysis, we additionally ran GO enrichment with the same settings on the marine control data set: aardvark, alpaca, camel, little brown bat, and David’s myotis bat. TRACCER has no significant terms with FDRs below 0.3 and is within expected levels of background noise. RERConverge, however, has ten significantly enriched GO-Terms with FDRs below 0.3, including “keratin filament” with a highly significant *P*-value of 3.3E−6 (fig. 4*E*, Supplementary S4 online). This raises the concern that the slight enrichment in the control *P*-value distribution produced by RERConverge (fig. 2*B*) may have a systematic trend; there are no known convergent changes to the integument of the control lineages and there is no immediate explanation for why the keratin gene set should be significantly enriched. In all, TRACCER yielded more significant genes, yet fewer significant GO terms, while maintaining a lower false-positive rate in the control GO-enrichment. This suggests improvements in both sensitivity and specificity.

### Test Case 2: Mammalian Longevity Convergence

**Fig. 5.**
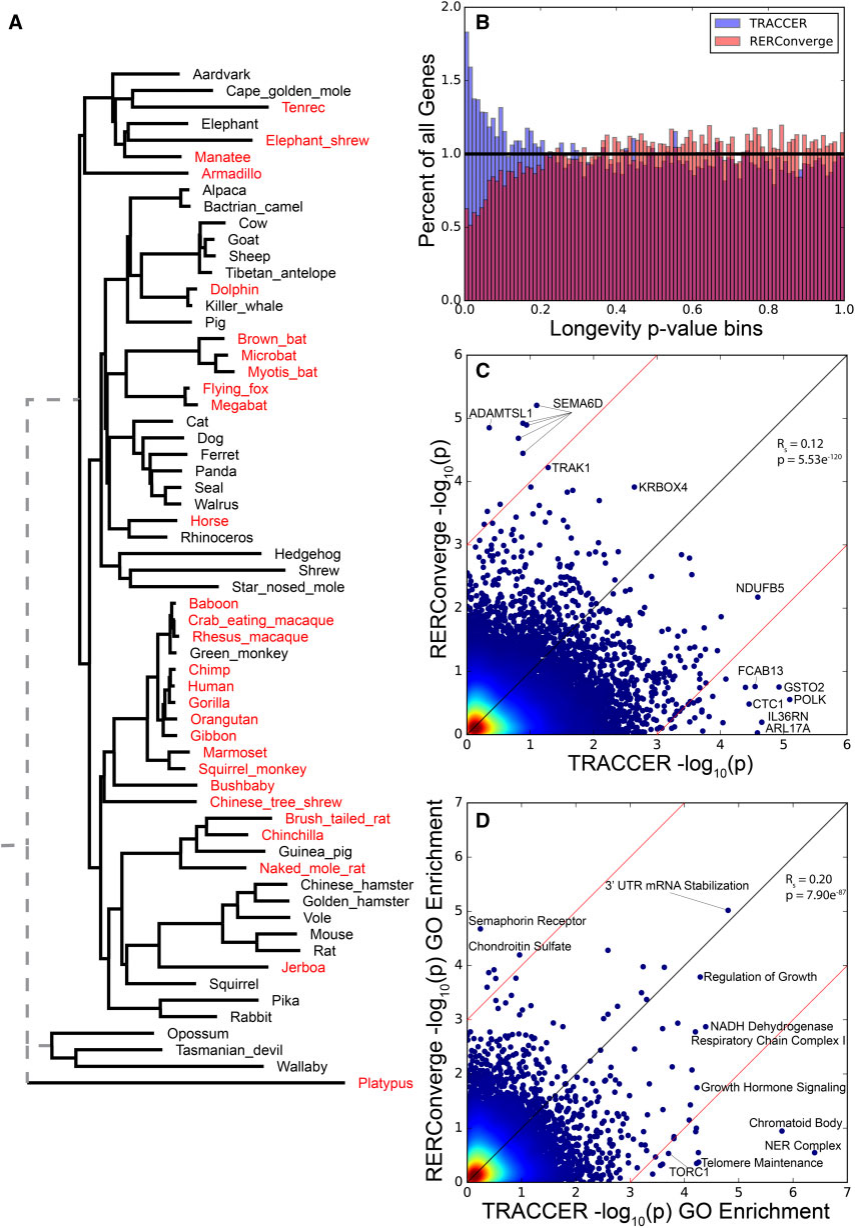
Comparison of TRACCER and RERConverge in convergence of mammalian longevity. (*A*) Mammalian phylogeny, highlighting long-lived species after correcting for body size. (*B*) Results from TRACCER (blue) are enriched at low *P*-values when analyzing for longevity convergence, while RERConverge (red) is depleted. (*C*) Concordance between TRACCER and RERConverge is significant (*P* = 5.53E−120) with notably different genes labeled. Spliceoforms are included. (*D*) Gene set enrichment between the two analyses shows significant concordance, but TRACCER has far more highly significant terms, including proofs of concept like Growth Signaling, TORC, Telomere Maintenance, and Nucleotide Excision Repair. These lend credence to the novel Chromatoid Body set as a promising set of candidates for future longevity investigations. In all, TRACCER yields 20 GO terms convergently evolving with longevity with FDRs below 0.1, while RERConverge has one. Both analyses agree on the significant 3′UTR-mediated mRNA stabilization class.

To assess TRACCER’s ability to parse convergent signals on a more enigmatic, but also more common trait, we applied it to longevity on the same mammalian phylogeny. Again, to compare how the inclusion of topology may augment convergence analyses, we matched the design to previous RERConverge studies (Kowalczyk et al. 2020). As in that study, the longevity trait was calculated as the second principal component of body size and maximum lifespan. These species are effectively “long-lived after correcting for the expected lifespan of their body-size.” However, TRACCER currently only operates on binary traits, so all positive values were treated as equivalently long-lived (fig. 5*A*). We compared these results directly to RERConverge operated with similar settings. Importantly, RERConverge has the functionality to analyze continuous traits; we ran it as binary nonetheless in order to keep the analyses as comparable as possible and gauge the benefit of integrating topology. TRACCER demonstrated substantial enrichment at low *P*-values, while RERConverge was depleted (fig. 5*B*). The concordance between the studies was significant, with a Spearman correlation 0.12 and *P*-value 5.53E−120 (fig. 5*C*). Again, many genes were dramatically shifted between the two analytic pipelines, as seen outside the red lines. Importantly, spliceoforms were included, so some proteins are listed multiple times to gain insight into exon-specific trends. To generalize the differences observed, we again performed SUMSTAT GO gene set enrichment, using only the most significant of each spliceoform when applicable (fig. 5*D*). TRACCER identifies “Positive Regulation of Growth” (*P *= 5.12E−5), “TORC1” (*P* = 1.9E−4), and “Telomere Maintenance” (*P* = 5.96E−5) as three compelling proofs of concept. These terms have obvious implications in the longevity literature (Foley et al. 2018; Papadopoli et al. 2019; Zhu et al. 2019) and lend credence to the other terms and genes identified in this analysis. Both analyses agree on the significant enrichment of “3′-UTR-mediated mRNA Stabilization,” which has previously been implicated in the evolution of longevity based on patterns of duplication (Doherty and de Magalhães 2016). However, TRACCER identifies the Nucleotide Excision Repair (NER) Complex (*P* = 4.00E−7) two orders of magnitude more significant than the other terms, and six orders greater than RERConverge scores it (*P* = 0.283). NER defects lead to cancer susceptibility and premature aging syndromes (Wong et al. 2020) and the core ERCC genes have been implicated in human longevity from SNP-SNP interactions (Dato et al. 2018). In parallel, two of the most significant individual genes, *POLK* and *ARL17A*, are both involved in broader NER activity. Interestingly, *POLK* deficient mice are normal in most regards but do exhibit reduced lifespan (Stancel et al. 2009) and *POLK* is one of the greatest differentially expressed repair genes when comparing mice to humans and naked mole rats—relatively long-lived mammals (MacRae et al. 2015). *ARL17A* expression is significantly associated with human longevity past the 90th percentile (Deelen et al. 2019). NER, and its core complex specifically, may be underappreciated in the evolution of longevity.

These proof-of-concept GO classes inspire confidence, and interest, in the highest-scoring genes that are not associated with any significant terms, suggesting they should be examined for implications in those systems. *GSTO2*, *ILL36RN*, *NDUFB5*, *EFCAB13*, and *CTC1* all have highly significant scores less than 5E−5 and FDRs less than 0.16 for evolving with longevity but are not present in the most significant GO terms. They may have unappreciated roles in growth regulation or DNA repair. The regions of variation and conservation in these genes and gene sets are exciting targets for future longevity investigations.

TRACCER results are also highly enriched for the “chromatoid body” gene set (*P* = 1.1E−6). This functional class of genes is much less studied in longevity than sets like TORC signaling and DNA repair but has intriguing ramifications and potential in the regulation of longevity. The chromatoid body is essential for maintaining male germ line quality, and its structure and function are demonstrably compromised with age (Santos et al. 2018). Of course, if the male germ line is compromised with age, it would be inherently difficult to select for longevity or the delayed maturation that reliably accompanies it. Many of these genes are validated in germ-line transposable element suppression but may be more pleiotropic than they first appear. They are potentially involved in a more general transposable element suppression to maintain genomic integrity with age. For instance, the most significant member of this set, *DDX25* (*P* = 3.7E−4), is an RNA helicase essential for spermatogenesis but is also broadly expressed in the brain and pituitary where it is poorly characterized. Transposable element suppression may have underappreciated and broad impacts on aging and longevity (Sturm et al. 2015). In all, TRACCER identifies substantially more significant genes and GO terms convergently evolving than comparable methods that remain agnostic to topology (Supplementary S5 and S6 online).

## Discussion

Convergence does not occur in isolation; it is inherently a comparative concept, and the various comparisons across a phylogeny are not all equivalent. Comparisons between those with more similar genetic contexts, and importantly, those encompassing trait changes, will be the most informative to unravel the mechanisms facilitating those trait changes. TRACCER is constructed around this concept in order to augment convergent relative rate analysis with the phylogenetic relationships. It builds upon previous work using Relative Evolutionary Rates to infer genomic systems facilitating instances of convergent evolution. TRACCER also includes a diagnostic mode, which is particularly useful for conceptualizing the shape of a convergent signal given a phylogeny of interest. The diagnostic mode is valuable when designing an experiment within a particular group and can guide sampling with an estimation of whether additional species will be worthwhile.

We compare TRACCER directly to RERConverge which uses a similar approach and was the first to use RER convergence across broad data sets (Chikina et al. 2016). We found RERConverge to be powerful and elegant, efficiently revealing significant genes and pathways underlying convergent trait evolution. We use it as a valuable benchmark for TRACCER as it is the most comparable method without factoring in the complexity of topology, though there are other subtle differences in the RER calculation details, including TRACCER’s log-transformed fold change RER and corrections for near-zero-length artifact branches. The differences in the results between the two approaches illuminate how the inclusion of phylogenetic relationships and the bypassing of ancestral state inferences may augment convergence analyses. To our knowledge, no other relative rate convergence analysis includes this set of features.

Instead of treating each branch as an independent and equivalent event, TRACCER’s pairwise approach makes comparisons in reference to each pair’s most recent common ancestor. This ensures that each comparison represents the same genetic context evolving over the same time frame, such that the calculated RERs are directly comparable. Each comparison can then be weighted based on the tree topology, without assumptions about ancestral states, and compensates for sampling biases. The rank or root transformations applied to both the RER differences and topological weights maintain the spirit of convergence; it must be a repeated signal, but some comparisons are more informative than others because of their phylogenetic interrelationships. To ignore topology is to leave behind these informative components of phylogenomic data that can refine the map between genotype and phenotype.

RERconverge implicated a number of genes in mammalian marine transitions despite their closest terrestrial relatives possessing similar signals and/or hinging upon the tenuous but required ancestral state assumptions (fig. 1). Flagging the pinniped ancestor as a marine would be a simple change that would dramatically change RERConverge results without altering the methodology; that branch represents one-seventh of the marine branches in the data set and can easily shift signals to and from significance across the genome. We repeated that ancestral state decision when using RERConverge to stay true to published results and to highlight the risks of such requirements. In the case of *PLCZ1*, a gene specific for sperm heads (Escoffier et al. 2016), a significant *P*-value led to the enrichment of multiple sperm GO terms, none of which have an a priori reason to be under purifying selection when adapting to an aquatic environment. To the contrary, scanning electron microscopy available for thirty-six ungulates and cetaceans suggests sperm-head morphology should be variable in the entire group (Meisner et al. 2005). In fact, the cetacean sperm are missing many of the structures present in the rest of the group, which would not suggest constraint at a genetic level. The loss of such structures is generally associated with a relaxation of selection (Lahti et al. 2009). Inferring ancestral states is often difficult, or even impossible, and discrepancies when assigning them can propagate errors to databases and downstream functional analyses in the field. Despite the difficulties and risks, most convergence analyses require it.

By utilizing the full wealth of information available from phylogenomics, TRACCER obviates the need to infer ancestral states and can detect a more refined set of elements than currently available RER approaches. Specificity was improved when applied to the marine mammal data set. This is particularly evident with the lower false discovery rates, flatter control distribution, and lack of significant hits in the control gene set enrichment as compared to RERConverge. With mammalian longevity, sensitivity was dramatically improved by TRACCER, with many more highly significant genes and gene sets being revealed. These included literature-validated controls like TORC signaling, telomere maintenance, and the NER complex as aspects convergently evolving to modulate mammalian longevity. When robust proofs-of-concept like these emerge, it increases confidence in the novel genes and terms arising alongside, like TRACCER’s highly significant “chromatoid body” GO Term. This gene set is currently only characterized in germ-line transposon suppression, but there is evidence it may be more general in maintaining genomic and epigenomic integrity. The top gene in this set, *DDX25*, is additionally expressed in the brain and pituitary, where maintaining genomic integrity could have organismal-wide effects via endocrine signaling. The specific highly significant genes that are absent from the enriched gene sets are strong candidates for targeted characterization and incorporation into those sets. For instance, *ZNF268* and *C10ORF25* are the most significant hits for marine transitions but have no characterized functions that match the top gene sets. Yet, human GWAS indicates *ZNF268* may be involved in eye shape (Hysi et al. 2020) and body-size ratios (Lotta et al. 2018), both of which are evolving with marine transitions. Similarly, *GSTO2*, *ILL36RN*, *NDUFB5*, *EFCAB13*, and *CTC1* are highly significant for convergently evolving with mammalian longevity but are not present in the most significant GO terms. Human GWAS indicates *GSTO2* may be involved in both the risk and age-of-onset of Alzheimer’s Disease (Allen et al. 2012). Intersecting data sets in these ways can generate specific testable hypotheses regarding the function of genes that would not be conceived of in isolation (Treaster et al. 2021).

Like all methods that analyze fixed gene trees, TRACCER inherits the limitations and inaccuracies of those inputs. The species phylogeny is the backbone of the calculation but may not accurately reflect the true phylogeny, or individual gene paths due to introgression, gene transfer, etc. Analyses are only as accurate as the input data; using a phylogeny with statistically unsound nodes or misattribution of terminal states will have consequences for the results. For TRACCER, these effects are comparable to those outlined in figure 2*B*; the magnitude of the consequences will be specific for the tree at hand, the nature of the error, and the proximity of the error to the greatest weighted trait-changes. TRACCER already accommodates discordance resulting in spurious near-zero-length branches, and future upgrades will account for broader tree discordance as informative events. In addition, an upgrade will track if any lineages are particularly incongruous with the rest of the data set, thereby catalyzing researchers to reassess that lineage’s information. A robust enough data set could be used to infer traits in uncharacterized lineages based solely on genomic signatures matching the characterized lineages. These planned upgrades will unfetter TRACCER from some of the limitations inherent to tree-based strategies and mitigate the inheritance of inaccuracies from those inputs. In TRACCER’s current form, its results already demonstrate that integrating topology into convergent relative rate analyses empowers comparative genomics to discover actionable targets to modulate traits of interest. The marine mammal genes and mammalian longevity genes newly identified by TRACCER are exciting candidates for targeted experimentation.

## Materials and Methods

**Fig. 6.**
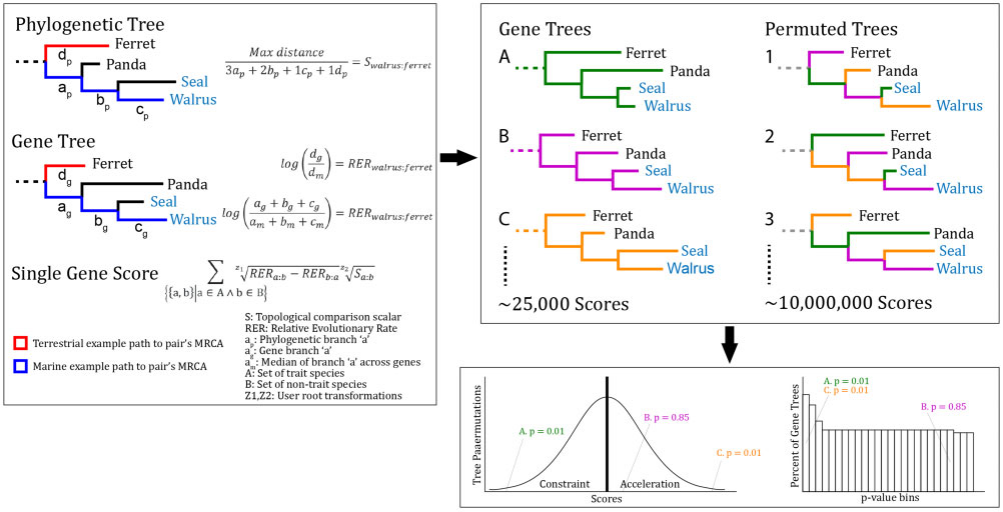
TRACCER example calculations. Binary trait status is assigned to each extant lineage. Each pairwise comparison of trait- and nontrait species has a scalar derived from the phylogenetic tree. For example, when comparing Walrus and Ferret, all branch lengths back to their MRCA on the phylogeny (a_p_, b_p_, …) are multiplied by the number of extant lineages sharing that branch (thus balancing for sampling biases) and summed. These are then inverted by taking the ratio with the maximum pairwise distance, such that the closest comparison has the greatest value. Using the gene tree, RERs for each comparison are derived as the log-ratio of distance to their MRCA (a_g_ + b_g_, …) to the median (a_m_ + b_m_, …) of that distance across all gene trees. Finally, the difference between RERs for each comparison, and the scalar for that comparison, are zth root transformed as chosen by the user, then summed across all comparisons to produce the gene score. To calculate significance, gene scores are then compared to those calculated from randomly permuted combinations of branches across all gene trees. Because the permutation score distribution may not be symmetrical, significance is determined in comparison only to those exhibiting the same pressures.

TRACCER operates on phylogenetic trees, the specifics of which can be decided by the experimenter as appropriate for their research. The only requirements are a species tree and a set of individual element trees fixed to the species tree topology. As rate changes are agnostic to the underlying units, the calculation can be performed on any continuous variable branch lengths. From the species tree, a scalar for each pairwise comparison is derived from the total distance between the two species, with each branch along that path weighted by the number lineages that share it (fig. 6). The scalars across the phylogeny are inverted, setting the furthest comparison to the lowest weight, and the closest comparison having the greatest weight. Two options are provided for an additional transformation: the Nth root as decided by the user, or a rank transformation across all comparisons. The analyses in this study used the rank transformation, with the closest species having the greatest rank. These transformations ensure that proximate comparisons are weighted more heavily without swamping the analysis, and distal comparisons are attenuated without being ignored.

The score for a given gene tree is the sum of all pairwise RER differences relative to their MRCA, each scaled by proximity and branch use on the species tree. To calculate RERs for these comparisons, TRACCER uses the medians of the distances across all genes for each lineage to their MRCA, ignoring zeroes and discordance artifacts, to derive a fold-change. Sequence divergence rates are guttered by a large number of zeroes due to slowly evolving short sequences without informative mutations. These zeroes result in a nonnormal distribution that makes the concept of “relative” rates meaningless unless they are excluded from this step of the calculation. Calculated relative rates are log-transformed, thereby balancing accelerated and decelerated fold-change relative rates; a one-tenth deceleration from 1 to 0.1 should be of the same importance as the ten-fold acceleration from 1 to 10, despite an order of magnitude difference in the raw differences. As would be expected, the data in our benchmarking investigations is log-normally distributed after removing the zeroes and artifacts.

For each combination of trait- and nontrait-bearing species, the log-RERs to their most recent common ancestor are subtracted to quantify differences and direction from their shared ancestral state. By focusing on differences, TRACCER can, for example, detect a relaxation of selection on a conserved element even if the rate is still below the background rate. The pairwise values across the tree are then Zth root transformed, or rank transformed, to correct for outliers while ensuring all comparisons have an impact. They are then multiplied by the scalar derived from the species tree to weigh comparisons by proximity and branch use. All pairwise values are then summed for a final gene score. These calculations are outlined in figure 6.

To ascertain a given score’s significance, a permutation test is performed to determine the background score distribution. As topology is fixed, branches can be shuffled between trees with millions of permutations and then scored as before. Importantly, when species are missing on a gene tree, it changes the shape of the possible scores and requires its own resampling to compare against. Finally, gene tree scores are compared to the permuted scores on the same tree topology to determine the percentage that are greater as a *P*-value. Gene trees exhibiting constraint are only compared to the resamples scoring below the median; those exhibiting acceleration are only compared to resamples above the median. FDRs are calculated with the Benjamini–Hochberg approach (Benjamini and Hochberg 1995).

Fixing trees to the same topology is required to directly compare branches between trees and to derive relative rates. This can be problematic for some analyses, because when a lineage is missing due to gene loss or lack of coverage, it may remove key branches and compromise the 1:1 mapping between trees. Because TRACCER performs comparisons in reference to their most recent common ancestor, lineages can still be consistently compared across genes even when other branches are missing. Though gene losses and missing coverage are indistinguishable, TRACCER still operates on these trees, but it performs the calculations within a compressed score-space specific for a topology without those lineages and uses a permuted score distribution specific for that topology to determine significance. Each unique topology is effectively an independent experiment. Future upgrades to TRACCER will allow missing data to be treated as informative if chosen by the user; if a lineage is missing due to documented gene loss, the information could, and should, be incorporated into the calculation.

Forcing the gene trees to a specific topology will introduce artifacts when the two disagree (Degnan and Rosenberg 2009), and it would be nonsensical to derive RERs directly from this discordance. Discordance over a larger distance could be detected by comparing fixed and unfixed gene trees, or sequence identity directly. Planned upgrades will factor this information as a complementary dimension of the convergence analysis, but they cannot be used for RERs directly. TRACCER does compensate for one form of discordance: closely related clades and slowly evolving genes often manifest extremely short, but nonzero, branch length artifacts. These artifacts are due to a lack of informative sites in a slowly evolving region. When a lineage has a single SNP while the rest of the clade is identical, the identical lineages will try to cluster. If those are not immediate sisters, it can yield short-nonzero branch lengths. These values can and should be transformed to zero as they represent no change; they are still identical to the ancestral state. TRACCER has an initial curation revealing the distribution of branch lengths to the user, indicating which branch lengths are likely artifacts, and allowing customization of the branch lengths the user wishes to transform. In our test data sets, these took the form of branches with lengths between 10^−4^ and 10^−7^. The exact values will vary based on the tree building software used, but with both PAML and IQ-Tree, in mammals, fish, and birds, we found 10^−4^ to be the right cutoff. While other analyses discard these values, or uses them without correction, TRACCER corrects these values to zero. However, should a gene-tree consist predominantly of these short values, TRACCER will discard it as evolving too slowly to be informative.

Trait distribution and topology is specific to every experiment and dramatically influences the possible score distributions. It may be impossible to differentiate noise from a true convergent signal if a data set is too simple. To determine if one’s experimental design and topology is sufficient to yield a convergent signal, and how robust that signal must be, TRACCER can be run in diagnostic mode. This feature uses only the species tree to generate millions of gene trees with Brownian motion and forces various convergent patterns upon them. These scores are compared to the strictly random trees to determine how often such scores occur by chance. This can inform experimental design and sampling strategies to optimize resources necessary to get an informative and accurate signal.

### Gene Set Enrichment

GO Terms for the mammalian data set were harvested from Ensembl. Terms with less than three members, or more than 500, were discarded as being uninformative or misleading in the context of set enrichment. The SUMSTAT approach was used on log-transformed *P*-values from each analysis, with an additional square root transformation to undermine outliers. Using *P*-values instead of scores is essential as it corrects for the variable score-space between trees with missing lineages. In short, the square-rooted, log transformed *P*-values for each gene in a set were summed and compared to a distribution of randomly sampled scores of the same set size to determine an enrichment *P*-value. Notably, we do not segregate genes into accelerated and constrained bins for performing enrichment, as other analyses have done. To handle spliceoforms, only the most significant convergent hit among them was included. FDRs are calculated with the Benjamini–Hochberg approach (Benjamini and Hochberg 1995). These FDRs are conservative, as GO Terms have varying degrees of overlap, such that the number of terms tested is far higher than the number of discrete concepts tested. At the gene level, the inclusion of spliceoforms makes the calculated FDRs roughly twice as conservative as they should be but provides potential insight into exon-specific convergence.

### RERConverge Settings

For marine and control convergence analysis, RERConverge settings were matched to their published protocols (Chikina et al. 2016). For the longevity analysis, the lineages with a positive second principle component of body size and longevity were flagged as bearing the trait (Kowalczyk et al. 2020). These species are effectively “long-lived after correcting for expected lifespan from their body-size.” We used RERConverge’s clade-weighting flag to automatically distribute their weight onto ancestral branches with clade = “all,” weighted = TRUE. When calculating residuals, we used the following settings: transform = “sqrt,” weighted = T, scale = T. Finally, to correlate the residuals with the binary phenotype, we used min.sp = 15, min.pos = 5, and weighted = “auto.” The minimum species correspond with the minimum species cutoffs used with TRACCER.

### TRACCER as Software

TRACCER’s permutation strategy can be computationally intensive, particularly if variable lineages are missing in many gene trees, as each tree shape will need its own set of permutations to determine its unique score space. The marine mammal data set included 62 lineages, covering 37,272 protein sequences, including spliceoforms, with 6,804 unique topologies. TRACCER runs on this data set size in 578 min with 14 CPUs to achieve a significance limit of 1.6E−7. These times are highly variable depending on the size of the data set and how many unique topologies there are. Importantly, additional topologies due to missing lineages are computationally inconsequential unless they include a highly significant convergence signal, which is rare by definition. The resources required are typically within the scope of any modern computational center, or even a well-equipped personal desktop. However, as the number of topologies can grow rapidly as more taxa become available, the programming may need efficiency optimizations in the future. For now, to scale the computational demand for different computing systems, TRACCER can automatically set the significance calculations to fit within a designated time window given the resources available. Alternatively, the user can demand the degree of precision and TRACCER will run until a certain number of resamples are performed, with time to completion estimates as it progresses.

TRACCER was written in Python with minimal dependencies for easy installation and execution in a variety of computing environments. Scripts, updates, and tutorials will be made publicly available at github.com/harris-fishlab/TRACCER and fishbonelab.org/.

## Supplementary Material

Supplementary data are available at *Molecular Biology and Evolution* online. 

## Supplementary Material

msab226_Supplementary_Data

## References

[msab226-B1] Ackermann M , Strimmer K. 2009. A general modular framework for gene set enrichment analysis. BMC Bioinformatics. 10:47. 19192285 10.1186/1471-2105-10-47 PMC2661051

[msab226-B2] Allen M , Zou F, Chai HS, Younkin CS, Miles R, Nair AA, Crook JE, Pankratz VS, Carrasquillo MM, Rowley CN, et al 2012. Glutathione S-transferase omega genes in Alzheimer and Parkinson disease risk, age-at-diagnosis and brain gene expression: an association study with mechanistic implications. Mol Neurodegener. 7:13. 22494505 10.1186/1750-1326-7-13 PMC3393625

[msab226-B3] Benjamini Y , Hochberg Y. 1995. Controlling the false discovery rate: a practical and powerful approach to multiple testing. J R Stat Soc Ser B Methodol. 57(1):289–300.

[msab226-B4] Berta A , Churchill M, Boessenecker RW. 2018. The origin and evolutionary biology of pinnipeds: seals, sea lions, and walruses. Annu Rev Earth Planet Sci. 46(1):203–228.

[msab226-B5] Chen X , Wang L, Hu B, Guo M, Barnard J, Zhu X. 2010. Pathway-based analysis for genome-wide association studies using supervised principal components. Genet Epidemiol. 34(7):716–724. 20842628 10.1002/gepi.20532 PMC3480088

[msab226-B6] Chikina M , Robinson JD, Clark NL. 2016. Hundreds of genes experienced convergent shifts in selective pressure in marine mammals. Mol Biol Evol. 33(9):2182–2192. 27329977 10.1093/molbev/msw112 PMC5854031

[msab226-B7] Currie A. 2013. Convergence as evidence. Br J Philos Sci. 64(4):763–786.

[msab226-B8] Daane JM , Auvinet J, Stoebenau A, Yergeau D, Harris MP, Detrich HW. 2020. Developmental constraint shaped genome evolution and erythrocyte loss in Antarctic fishes following paleoclimate change. PLoS Genet. 16(10):e1009173. 33108368 10.1371/journal.pgen.1009173 PMC7660546

[msab226-B9] Dato S , Soerensen M, Rango FD, Rose G, Christensen K, Christiansen L, Passarino G. 2018. The genetic component of human longevity: new insights from the analysis of pathway-based SNP-SNP interactions. Aging Cell. 17(3):e12755. 29577582 10.1111/acel.12755 PMC5946073

[msab226-B10] Deelen J , Evans DS, Arking DE, Tesi N, Nygaard M, Liu X, Wojczynski MK, Biggs ML, van der Spek A, Atzmon G, et al 2019. A meta-analysis of genome-wide association studies identifies multiple longevity genes. Nat Commun. 10(1):3669. 31413261 10.1038/s41467-019-11558-2 PMC6694136

[msab226-B11] Degnan JH , Rosenberg NA. 2009. Gene tree discordance, phylogenetic inference and the multispecies coalescent. Trends Ecol Evol. 24(6):332–340. 19307040 10.1016/j.tree.2009.01.009

[msab226-B12] Doherty A , de Magalhães JP. 2016. Has gene duplication impacted the evolution of Eutherian longevity? Aging Cell. 15(5):978–980. 27378378 10.1111/acel.12503 PMC5013011

[msab226-B13] Downing Meisner A , Klaus AV, O'Leary MA. 2005. Sperm head morphology in 36 species of artiodactylans, perissodactylans, and cetaceans (Mammalia). J Morphol. 263(2):179–202. 15593320 10.1002/jmor.10297

[msab226-B14] Echave J , Spielman SJ, Wilke CO. 2016. Causes of evolutionary rate variation among protein sites. Nat Rev Genet. 17(2):109–121. 26781812 10.1038/nrg.2015.18 PMC4724262

[msab226-B15] Escoffier J , Lee HC, Yassine S, Zouari R, Martinez G, Karaouzène T, Coutton C, Kherraf Z, Halouani L, Triki C, et al 2016. Homozygous mutation of *PLCZ1* leads to defective human oocyte activation and infertility that is not rescued by the WW-binding protein PAWP. Hum Mol Genet. 25(5):878–891. 26721930 10.1093/hmg/ddv617 PMC4754041

[msab226-B16] Foley NM , Hughes GM, Huang Z, Clarke M, Jebb D, Whelan CV, Petit EJ, Touzalin F, Farcy O, Jones G, et al 2018. Growing old, yet staying young: the role of telomeres in bats’ exceptional longevity. Sci Adv. 4(2):eaao0926. 29441358 10.1126/sciadv.aao0926 PMC5810611

[msab226-B17] Gould SJ. 2002. The structure of evolutionary theory. Cambridge (MA): Harvard University Press.

[msab226-B18] Hayden S , Bekaert M, Crider TA, Mariani S, Murphy WJ, Teeling EC. 2010. Ecological adaptation determines functional mammalian olfactory subgenomes. Genome Res. 20(1):1–9. 19952139 10.1101/gr.099416.109 PMC2798820

[msab226-B19] Hiller M , Schaar BT, Indjeian VB, Kingsley DM, Hagey LR, Bejerano G. 2012. A “forward genomics” approach links genotype to phenotype using independent phenotypic losses among related species. Cell Rep. 2(4):817–823. 23022484 10.1016/j.celrep.2012.08.032 PMC3572205

[msab226-B20] Hysi PG , Choquet H, Khawaja AP, Wojciechowski R, Tedja MS, Yin J, Simcoe MJ, Patasova K, Mahroo OA, Thai KK, et al; 23andMe Inc. 2020. Meta-analysis of 542,934 subjects of European ancestry identifies new genes and mechanisms predisposing to refractive error and myopia. Nat Genet. 52(4):401–407. 32231278 10.1038/s41588-020-0599-0 PMC7145443

[msab226-B21] Katzman S , Kern AD, Bejerano G, Fewell G, Fulton L, Wilson RK, Salama SR, Haussler D. 2007. Human genome ultraconserved elements are ultraselected. Science 317(5840):915. 17702936 10.1126/science.1142430

[msab226-B22] Kishida T , Thewissen J, Hayakawa T, Imai H, Agata K. 2015. Aquatic adaptation and the evolution of smell and taste in whales. Zool Lett. 1:9. 10.1186/s40851-014-0002-z PMC4604112 26605054

[msab226-B23] Koretsky I , Barnes L. 2003. Origins and relationships of pinnipeds, and the concepts of monophyly versus diphyly. J Vertebr Paleontol. 23:69A–69A.

[msab226-B24] Kowalczyk A , Partha R, Clark NL, Chikina M. 2020. Pan-mammalian analysis of molecular constraints underlying extended lifespan. ELife 9:e51089. 32043462 10.7554/eLife.51089 PMC7012612

[msab226-B25] Lahti DC , Johnson NA, Ajie BC, Otto SP, Hendry AP, Blumstein DT, Coss RG, Donohue K, Foster SA. 2009. Relaxed selection in the wild. Trends Ecol Evol. 24(9):487–496. 19500875 10.1016/j.tree.2009.03.010

[msab226-B26] Losos JB. 2011. Convergence, adaptation, and constraint. Evolution 65(7):1827–1840. 21729041 10.1111/j.1558-5646.2011.01289.x

[msab226-B27] Lotta LA , Wittemans LBL, Zuber V, Stewart ID, Sharp SJ, Luan J, Day FR, Li C, Bowker N, Cai L, et al 2018. Association of genetic variants related to gluteofemoral vs abdominal fat distribution with type 2 diabetes, coronary disease, and cardiovascular risk factors. JAMA 320(24):2553–2563. 30575882 10.1001/jama.2018.19329 PMC6583513

[msab226-B28] MacRae SL , Croken MM, Calder RB, Aliper A, Milholland B, White RR, Zhavoronkov A, Gladyshev VN, Seluanov A, Gorbunova V, et al 2015. DNA repair in species with extreme lifespan differences. Aging (Albany NY). 7(12):1171–1182. 26729707 10.18632/aging.100866 PMC4712340

[msab226-B29] Marcovitz A , Turakhia Y, Chen HI, Gloudemans M, Braun BA, Wang H, Bejerano G. 2019. A functional enrichment test for molecular convergent evolution finds a clear protein-coding signal in echolocating bats and whales. Proc Natl Acad Sci U S A. 116(42):21094–21103. 31570615 10.1073/pnas.1818532116 PMC6800341

[msab226-B30] Mundry R. 2014. Statistical issues and assumptions of phylogenetic generalized least squares. In: Garamszegi L.Z., editor. Modern phylogenetic comparative methods and their application in evolutionary biology: concepts and practice. Berlin, Heidelberg: Springer. p. 131–153.

[msab226-B31] Muraco H , Kuczaj SAII. 2015. Conceptive estrus behavior in three bottlenose dolphins (*Tursiops truncatus*). *Anim Behav Cogn*. 2:30–48.

[msab226-B32] Naser-Khdour S , Minh BQ, Zhang W, Stone EA, Lanfear R. 2019. The prevalence and impact of model violations in phylogenetic analysis. Genome Biol Evol. 11(12):3341–3352. 31536115 10.1093/gbe/evz193 PMC6893154

[msab226-B33] Palazzo AF , Gregory TR. 2014. The case for junk DNA. PLoS Genet. 10(5):e1004351. 24809441 10.1371/journal.pgen.1004351 PMC4014423

[msab226-B34] Papadopoli D , Boulay K, Kazak L, Pollak M, Mallette F, Topisirovic I, Hulea L. 2019. *mTOR* as a central regulator of lifespan and aging. F1000Research. 8(F1000 Faculty Rev):998. 10.12688/f1000research.17196.1 PMC6611156 31316753

[msab226-B35] Parker J , Tsagkogeorga G, Cotton JA, Liu Y, Provero P, Stupka E, Rossiter SJ. 2013. Genome-wide signatures of convergent evolution in echolocating mammals. Nature 502(7470):228–231. 24005325 10.1038/nature12511 PMC3836225

[msab226-B36] Partha R , Chauhan BK, Ferreira Z, Robinson JD, Lathrop K, Nischal KK, Chikina M, Clark NL. 2017. Subterranean mammals show convergent regression in ocular genes and enhancers, along with adaptation to tunneling. ELife 6:e25884. 29035697 10.7554/eLife.25884 PMC5643096

[msab226-B37] Pearce T. 2012. Convergence and parallelism in evolution: a Neo-Gouldian account. Br J Philos Sci. 63(2):429–448.

[msab226-B38] Pollard KS , Salama SR, King B, Kern AD, Dreszer T, Katzman S, Siepel A, Pedersen JS, Bejerano G, Baertsch R, et al 2006. Forces shaping the fastest evolving regions in the human genome. PLoS Genet. 2(10):e168. 17040131 10.1371/journal.pgen.0020168 PMC1599772

[msab226-B39] Prudent X , Parra G, Schwede P, Roscito JG, Hiller M. 2016. Controlling for phylogenetic relatedness and evolutionary rates improves the discovery of associations between species’ phenotypic and genomic differences. Mol Biol Evol. 33(8):2135–2150. 27222536 10.1093/molbev/msw098 PMC4948712

[msab226-B40] Revell LJ , Harmon LJ, Collar DC. 2008. Phylogenetic signal, evolutionary process, and rate. Syst Biol. 57(4):591–601. 18709597 10.1080/10635150802302427

[msab226-B41] Rosenbloom KR , Armstrong J, Barber GP, Casper J, Clawson H, Diekhans M, Dreszer TR, Fujita PA, Guruvadoo L, Haeussler M, et al 2015. The UCSC Genome Browser database: 2015 update. Nucleic Acids Res. 43(Database issue):D670–D681. 25428374 10.1093/nar/gku1177 PMC4383971

[msab226-B42] Rosenblum EB , Parent CE, Brandt EE. 2014. The molecular basis of phenotypic convergence. Annu Rev Ecol Evol Syst. 45(1):203–226.

[msab226-B43] Santos EG , Silva MA, Amorim RP, Giordano L, de S, Silva D, de S, Rasmussen LT, Peruquetti RL. 2018. Aging and chromatoid body assembly: are these two physiological events linked? Exp Biol Med (Maywood). 243(11):917–925. 29958504 10.1177/1535370218784871 PMC6108056

[msab226-B44] Schermerhorn T. 2013. Normal glucose metabolism in carnivores overlaps with diabetes pathology in non-carnivores. Front Endocrinol (Lausanne). 4:188. 24348462 10.3389/fendo.2013.00188 PMC3847661

[msab226-B45] Slater GJ , Harmon LJ, Alfaro ME. 2012. Integrating fossils with molecular phylogenies improves inference of trait evolution. Evolution 66(12):3931–3944. 23206147 10.1111/j.1558-5646.2012.01723.x

[msab226-B46] Stancel JNK , McDaniel LD, Velasco S, Richardson J, Guo C, Friedberg EC. 2009. *Polk* mutant mice have a spontaneous mutator phenotype. DNA Repair (Amst). 8(12):1355–1362. 19783230 10.1016/j.dnarep.2009.09.003 PMC2787749

[msab226-B47] Sturm Á , Ivics Z, Vellai T. 2015. The mechanism of ageing: primary role of transposable elements in genome disintegration. Cell Mol Life Sci. 72(10):1839–1847. 25837999 10.1007/s00018-015-1896-0 PMC11113528

[msab226-B48] Tegoni M , Pelosi P, Vincent F, Silvia S, Campanacci V, Grolli S, Ramoni R, Cambillau C. 2000. Mammalian odorant binding proteins. Biochim Biophys Acta BBA - Protein Struct Mol Enzymol. 1482(1-2):229–240. 10.1016/s0167-4838(00)00167-9 11058764

[msab226-B49] Tintle NL , Borchers B, Brown M, Bekmetjev A. 2009. Comparing gene set analysis methods on single-nucleotide polymorphism data from Genetic Analysis Workshop 16. BMC Proc. 3 Suppl 7:S96. 20018093 10.1186/1753-6561-3-s7-s96 PMC2796000

[msab226-B50] Treaster S , Karasik D, Harris MP. 2021. Footprints in the sand: deep taxonomic comparisons in vertebrate genomics to unveil the genetic programs of human longevity. Front Genet. 12:678073. 34163529 10.3389/fgene.2021.678073 PMC8215702

[msab226-B51] Venn-Watson S. 2014. Dolphins and diabetes: applying one health for breakthrough discoveries. Front Endocrinol (Lausanne). 5:227. 25566195 10.3389/fendo.2014.00227 PMC4273662

[msab226-B52] Wong A , Kieu T, Robbins PD. 2020. The *Ercc1*-/Δ mouse model of accelerated senescence and aging for identification and testing of novel senotherapeutic interventions. Aging (Albany NY). 12(24):24481–24483. 33353886 10.18632/aging.202321 PMC7803498

[msab226-B53] Yu L , Jin W, Wang J, Zhang X, Chen M, Zhu Z, Lee H, Lee M, Zhang Y. 2010. Characterization of *TRPC2*, an essential genetic component of VNS chemoreception, provides insights into the evolution of pheromonal olfaction in secondary-adapted marine mammals. Mol Biol Evol. 27(7):1467–1477. 20142439 10.1093/molbev/msq027

[msab226-B54] Zhu Y , Liu X, Ding X, Wang F, Geng X. 2019. Telomere and its role in the aging pathways: telomere shortening, cell senescence and mitochondria dysfunction. Biogerontology 20(1):1–16. 30229407 10.1007/s10522-018-9769-1

[msab226-B55] Zou Z , Zhang J. 2015. No genome-wide protein sequence convergence for echolocation. Mol Biol Evol. 32(5):1237–1241. 25631925 10.1093/molbev/msv014 PMC4408410

